# Synthetic Aperture Radar Processing Approach for Simultaneous Target Detection and Image Formation

**DOI:** 10.3390/s18103377

**Published:** 2018-10-10

**Authors:** Jifang Pei, Yulin Huang, Weibo Huo, Yuxuan Miao, Yin Zhang, Jianyu Yang

**Affiliations:** Department of Electrical Engineering, University of Electronic Science and Technology of China, No. 2006, Xiyuan Ave., West Hi-Tech Zone, Chengdu 611731, China; hwbuyi@163.com (W.H.); yuxuanm_work@126.com (Y.M.); yinzhang@uestc.edu.cn (Y.Z.); jyyang@uestc.edu.cn (J.Y.)

**Keywords:** synthetic aperture radar, target detection, image formation, back-projection, visua saliency

## Abstract

Finding out interested targets from synthetic aperture radar (SAR) imagery is an attractive but challenging problem in SAR application. Traditional target detection is independent on SAR imaging process, which is purposeless and unnecessary. Hence, a new SAR processing approach for simultaneous target detection and image formation is proposed in this paper. This approach is based on SAR imagery formation in time domain and human visual saliency detection. First, a series of sub-aperture SAR images with resolutions from low to high are generated by the time domain SAR imaging method. Then, those multiresolution SAR images are detected by the visual saliency processing, and the corresponding intermediate saliency maps are obtained. The saliency maps are accumulated until the result with a sufficient confidence level. After some screening operations, the target regions on the imaging scene are located, and only these regions are focused with full aperture integration. Finally, we can get the SAR imagery with high-resolution detected target regions but low-resolution clutter background. Experimental results have shown the superiority of the proposed approach for simultaneous target detection and image formation.

## 1. Introduction

Synthetic aperture radar (SAR) can obtain high-resolution microwave images, with day or night operation capability [1,2,3]. And it is scarcely affected by the atmospheric and weather conditions. As an important modern radar system, it offers abundant and distinctive reconnaissance, surveillance and remote sensing data for both military and civilian applications [4,5].

Nowadays, people are interested in not only imaging processing but also interpretation or recognition of the real-world targets from radar imagery [6,7,8,9,10]. The general framework of an end-to-end SAR interpretation or automatic target recognition (ATR) system has three stages with a hierarchical processing [11,12,13]: detection, discrimination, and classification. As an important stage in SAR ATR system, detection of the real-world targets from SAR imagery is one of the most challenging research directions in SAR application [11]. Target detection isolates the regions of interest (ROI) from the SAR images by decision rules, and localizes those regions in the image where a potential target is likely to be present [14]. Target detection is very useful to discover the military or civilian targets, such as tanks, missile launching vehicle, ships and oil spill, from large-scale-scene SAR images. And it also directly impacts the succeeding process in SAR ATR system.

A large number of SAR target detection algorithms have been proposed in recent years, and those algorithms can generally be classified into two distinct categories [11]: single feature-based and multiple feature-based. Single feature-based approach is the most common but simple methodology in SAR image target detection. The widely used feature for this approach is the pixel brightness or radar cross section (RCS). Constant false alarm rate (CFAR) method is the most popular single feature-based detection method [15]. It adopts a sliding window structure and compares the SAR image pixel under test with a threshold calculated by its surroundings with this window. Based on this strategy, many variants of CFAR method have been proposed, such as cell-averaging CFAR (CA-CFAR) [16], order statistics CFAR (OS-CFAR) [17] and two-parameter CFAR (TP-CFAR) [18], which can perform well in practice. However, these algorithms are dependent on the prior knowledge of the imaging background, thus the detection results are often affected by the accuracy of the clutter modeling. In contrast, multiple feature-based methods try to fuse two or more features to make the final detection [19,20,21]. Therefore, this taxon can incorporate additional features besides the pixel brightness, such as fractal dimension, space scaling features, time-frequency features, etc. Multiple feature-based taxon can circumvent the drawback of the single feature-based one to some extent. However, the choice and extraction of the multiple features from the SAR image will incur additional complexity. Therefore, a tradeoff between the detection performance and computational complexity should be carefully taken into account.

Generally, almost all the existing target detection methods are carried out on the obtained high-resolution SAR images. In other words, the target detection stage is independent on the SAR imagery formation. In reality, there is a flaw in such a framework with sequential operations. The target detection stage must be proceeded after the imaging processing. However, in practice, only the ROIs on the imaging scenes, such as the regions containing vehicles, ships, buildings, etc., could be concerned, while other clutter regions are often unwanted and negligible. Therefore, high resolution imaging for the whole reconnaissance scene before target detection is purposeless and unnecessary. It is desirable to obtain a framework that can detect the ROIs during SAR imaging processing such that those regions are focused with high-resolution processing. Meanwhile, the remaining clutter regions are ignored or focused with low-resolution processing.

In this paper, we propose a new SAR processing approach which can simultaneously carry out target detection and image formation. First, a series of multiresolution SAR images are generated by time domain SAR imaging algorithm. Then, those multiresolution SAR images are detected by the visual saliency method, and the corresponding intermediate saliency maps with different confidence levels are obtained. The saliency maps are accumulated until the result with a sufficient confidence level. After screening, the ROIs on the imaging scene are located, and those regions will be focused with full-aperture integration. Finally, the output of the proposed SAR processing approach is the imagery with high-resolution target detection results but low-resolution clutter background.

The remainder of this paper consists of the following sections. Section 2 details the proposed SAR processing approach, and the experiments are carried out in Section 3 to evaluate the proposed approach. Conclusions are given in Section 4.

## 2. Proposed SAR Processing Approach

The capability of human visual system to find out the targets of interest is effective and reliable [22,23]. It has been proved that the human visual attention system can stare at prominent targets of interest in a scene [22]. It is well known that our eyes have a low resolution from a distance but have a good resolution when close to the scene. When we keep our eyes on a scene from far to near, the visual attention system keeps on detecting the interested targets from the images that the visual system generates in the brain with resolutions from low to high. In this process, the impression of the prominent and noticeable targets attracting much of our attention will continuously strengthen in our brain until those targets are regarded as what we are looking for.

**Figure 1:**
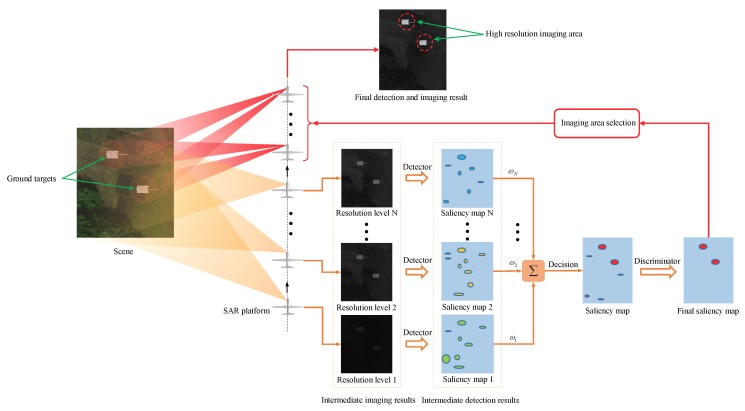
Basic scheme of proposed SAR target detection and imagery formation approach.

Inspired by this rationale, a novel SAR processing approach for simultaneous target detection and image formation is proposed based on the time domain SAR imaging [24] and visual saliency detection [25]. The time domain SAR imaging method with spotlight pattern generates a series of sub-aperture SAR images with resolutions from low to high, which is similar to the human visual system observing a scene from far to near. Meanwhile, as the human visual sweeping the visual field and finding out the prominent objects, the visual saliency algorithm detects the multiresolution SAR images, and obtains the corresponding intermediate saliency maps. Those intermediate saliency maps are accumulated until the results with a sufficient confidence level. After discriminating, the ROIs on the imaging scene are located, and those regions will be focused with full-aperture integration. Finally, we can obtain the SAR imagery with high-resolution target detection regions. The basic scheme of the proposed SAR target detection and imagery formation approach is illustrated in Figure 1.

Since the basic scheme of the proposed approach has been modeled, next we will discuss the implementation of the proposed approach.

### 2.1. Time Domain SAR Imagery Formation

While some SAR imaging methods in time domain exist, the most widely used method for implementation is the back-projection (BP) algorithm [24]. BP algorithm for SAR image reconstruction originates from the computed tomography imaging techniques [26]. A distinct advantage of the BP algorithm is the ability to form SAR image under arbitrary trajectory of the platform. Besides, it can straightforwardly generate the intermediate multiresolution SAR images along the cross-range, which is appropriate for the proposed approach. Recently, BP has been implemented on graphic processing units [27], and several fast BP methods also have been proposed to reduce the computational complexity [28,29]. For simplicity, only the classical BP algorithm will be introduced in the following.

Suppose the SAR sensor travels along a flight path and transmits the signal $s\left(t\right)$ with the spotlight pattern. The spatial location of a point on the discrete scene is $\left(x_{i},r_{j},0\right)$, where $x_{i}$ and $r_{j}$ denote the coordinates of the cross range and range, respectively. The location of the radar platform at time η is $\left(x\left(\eta \right),-R_{0},H\right)$, and the echo can be expressed as

(1) $$s_{r}\left(t,\eta \right)=\sum_{i,j}\sigma_{ij}s\left(t-\frac{2\sqrt{{\left(r_{j}+R_{0}\right)}^{2}+{\left(x_{i}-x\left(\eta \right)\right)}^{2}+H^{2}}}{c}\right)$$

where $\sigma_{ij}$ is related to the RCS of the point $\left(x_{i},r_{j},0\right)$. Thus the SAR imagery formation can be represented as

(2) $$\begin{array}{c} I\left(x_{i},r_{j}\right)=\;\int \int \;s_{r}\left(t,\eta \right)s^{*}\left(t-\frac{2\sqrt{{\left(r_{j}+R_{0}\right)}^{2}+{\left(x_{i}-x\left(\eta \right)\right)}^{2}+H^{2}}}{c}\right)dtd\eta \\ =\;\int \int \;s_{r}\left(t,\eta \right)s^{*}\left(t-t_{ij}\left(\eta \right)\right)dtd\eta \end{array}$$

where $s^{*}\left(t-t_{ij}\left(\eta \right)\right)$ is the matching filter of the point $\left(x_{i},r_{j},0\right)$. Because the range matching filter for each point are constant, the imaging processing can be decomposed into range compression and back projection. The signal after range compression can be expressed as

(3) $$\begin{array}{c} s_{M}\left(t,\eta \right)=s_{r}\left(t,\eta \right)⊗s^{*}\left(-t\right)\\ =\int s_{r}\left(\tau ,\eta \right)s^{*}\left(\tau -t\right)d\tau \end{array}$$

where $s^{*}\left(-t\right)$ denotes the range matching filter. After range compression, back projection starts to focus the echo date to generate low to high resolution SAR images, which can be used for the target detection processing. This imaging process can be represented as

(4) $$I\left(x_{i},r_{j}\right)=\int s_{M}\left(t_{ij}\left(\eta \right),\eta \right)d\eta$$

### 2.2. Visual Saliency Detection

The visual saliency method is employed to detect the multiresolution SAR images generated by BP, and to obtain their corresponding intermediate saliency maps in the proposed approach. There are many detection methods based on visual saliency principle [30,31,32]. In this implementation, the saliency detection method based on spectral residual [25] is utilized because of its effectiveness, feature independence and without other forms of prior knowledge of the targets, which is applicable to detect the ROIs from multiresolution SAR images.

From the perspective of information theory, the image information can be decomposed into the innovation and the prior knowledge. The innovation means the novelty part, and the prior knowledge denotes the redundant information should be suppressed during target detection. The saliency detection method based on spectral residual analyzes the log spectrum of the SAR image and calculate the spectral residual. Then the spectral residual is transformed into spatial domain, thus the saliency map is obtained.

Given an input SAR image $I_{k}\left(x,r\right)$ with resolution level *k*, its spectrum can be calculated by

(5) $$I_{k}\left(f_{x},f_{r}\right)=F\left[I_{k}\left(x,r\right)\right]$$

where $F\left(\cdot \right)$ denotes the two dimensional Fourier transform. Thus the corresponding amplitude spectrum and phase spectrum can be respectively expressed as

(6) $$A_{k}\left(f_{x},f_{r}\right)=A\left[I_{k}\left(f_{x},f_{r}\right)\right]$$

(7) $$P_{k}\left(f_{x},f_{r}\right)=P\left[I_{k}\left(f_{x},f_{r}\right)\right]$$

where $A\left(\cdot \right)$ and $P\left(\cdot \right)$ denote taking the amplitude and phase of the input, respectively. Then the log spectrum of the image can be obtained by

(8) $$L_{k}\left(f_{x},f_{r}\right)=\ln\left[A_{k}\left(f_{x},f_{r}\right)\right]$$

Thus, the spectral residual can be calculated by

(9) $$R_{k}\left(f_{x},f_{r}\right)=L_{k}\left(f_{x},f_{r}\right)-h\left(f_{x},f_{r}\right)*L_{k}\left(f_{x},f_{r}\right)$$

where $h\left(f_{x},f_{r}\right)$ is a local average filter defined as an n×n matrix:

(10) $$h\left(f_{x},f_{r}\right)=\frac{1}{n^{2}}\left(\begin{array}{cccc} 1 & 1 & \cdots & 1\\ 1 & 1 & \cdots & 1\\ \vdots & \vdots & \ddots & \vdots \\ 1 & 1 & \cdots & 1 \end{array}\right)$$

After two dimensional inverse Fourier transform and Gaussian filtering, the saliency map can be obtained in spatial domain:

(11) $$S_{k}\left(x,r\right)=g\left(x,r\right)*F^{-1}{\left[\exp\left(R_{k}\left(f_{x},f_{r}\right)+P_{k}\left(f_{x},f_{r}\right)\right)\right]}^{2}$$

where $F^{-1}\left(\cdot \right)$ denotes the two dimensional inverse Fourier transform, $g\left(x,r\right)$ is the Gaussian filter defined by

(12) $$g\left(x,r\right)=\frac{1}{2\pi \nu^{2}}\exp\left(-\frac{x^{2}+r^{2}}{2\nu^{2}}\right)$$

and ν is the filter parameter.

### 2.3. Saliency Accumulation and Decision

With the BP generating a series of multiresolution SAR images, the visual saliency detection method obtains their corresponding saliency maps. Because the intermediate SAR images are with resolution levels from low to high, the detection results on the saliency maps also have different confidence levels. The intermediate SAR image integrated from a short sub-aperture has low resolution in cross range. Hence, the visual quality of this image is poor, so the detection result is also with a low confidence level, and vice versa.

In order to get an accurate detection result during SAR imaging, a reliable way is accumulating those intermediate saliency maps until the results with a sufficient confidence level. Weighted summation of the intermediate saliency maps is a simple and effective method to make accumulation. Given a series of the intermediate saliency maps $\left\{S_{k}\left(x,r\right),k=1,2,\cdots ,N\right\}$, the saliency accumulation can be calculated by

(13) $$S_{l}^{\prime }\left(x,r\right)=\sum_{k=1}^{l}\omega_{k}S_{k}\left(x,r\right)$$

where ${S_{l}}^{\prime }\left(x,r\right)$ is the lth saliency accumulation result, and $\omega_{k}>0$ is the weight of the $S_{k}\left(x,r\right)$. Generally, the value of $\omega_{k}$ is positively related to the resolution level of $I_{k}\left(x,r\right)$, i.e., the higher the resolution level of $I_{k}\left(x,r\right)$, the higher value its weight $\omega_{k}$ has.

With the saliency accumulating, the target regions decision from the accumulated saliency map is also carried out by a threshold segmentation. The target regions decision is obtained by

(14) $$O_{l}\left(x,r\right)=\left\{\begin{array}{c} 1,\mathrm{if}{S^{\prime }}_{l}\left(x,r\right)>\delta \max\left({S^{\prime }}_{l}\left(x,r\right)\right)\\ 0,\mathrm{otherwise} \end{array}\right.$$

where $\max\left({S^{\prime }}_{l}\left(x,r\right)\right)$ is the maximum of the accumulated saliency map, and δ is a parameter to make a trade-off between the neglect of targets and false alarm.

As the decision processes continue, we can get a series of $\left\{O_{l}\left(x,r\right),l=1,2,\cdots ,L,L\leq N\right\}$ containing the decision results. Then a terminal criterion is used to stop this iteration: if there are *m* successive decision results with the same target regions, they are of a sufficient confidence level.

### 2.4. Final Detection and ROIs Imaging

Although the decision result has been obtained by the above processing, there may be some false alarm regions on the decision result. Thus, some discriminating operations should be carried out on the decision result. The geometrical features of the target regions are utilized to remove the false alarms. For simplicity, we use two geometrical features here for discriminating. The first one is the area of the target region: if $a\in \left[a_{\min},a_{\max}\right]$, the region under decision is labeled as a target, otherwise, it is a false alarm region, where *a* is the sum of the region pixels under decision, $a_{\min}$ and $a_{\max}$ are the minimum and maximum sizes of the actual target region on the SAR image, respectively. The other one is the length of the axes of the target region: if $b\in \left[\beta b_{\max},b_{\max}\right]$ and $b^{\prime }\in \left[b_{\min},b_{\max}\right]$, the region under decision is regarded as a target, otherwise, it is a false alarm, where *b* is the length of the major axis of the ellipse that has the same normalized second central moments as the region under decision, $b^{\prime }$ is the length of the minor axis of the ellipse that has the same normalized second central moments as the region under decision, β is a scaling factor, $b_{\min}$ and $b_{\max}$ is the minimum and maximum lengths of the actual target region on the SAR image, respectively.

The ROIs on the imaging scene are located after discriminating. Hence, those regions can be focused with full aperture integration. Finally, the SAR imagery with high-resolution target detection regions is obtained.

**Figure 2:**
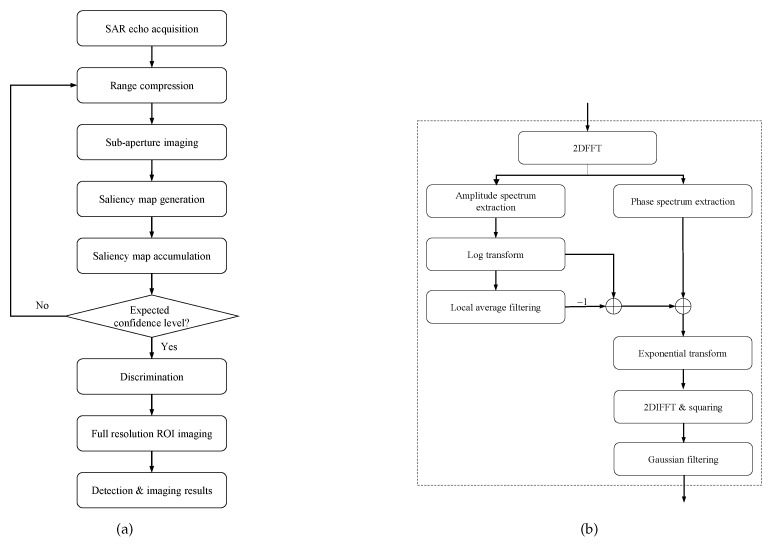
Process of proposed SAR processing approach implementation. (**a**) whole process of proposed approach and (**b**) saliency map generation module.

So far, the implementation of the proposed SAR processing approach for simultaneous target detection and image formation has been described. The whole implementation process and its saliency map generation module are summarized in Figure 2.

Now, we analyze the computational complexity of the proposed SAR processing approach. Suppose the size of the SAR imagery is M×M, the number of the echoes along the cross range is also *M*, and there are *K* echoes along the cross range for sub-aperture integration. Besides, there are *l* iterations for target detection, the number of the ROIs on the imaging scene is *p*, and the size of each ROI is q×q.

The computational complexity of the sub-aperture integration is $O\left(KM^{2}\right)$. The computational complexity for each saliency map generation is $2\cdot O\left(M^{2}\log_{2}M\right)$, so the total complexity of *l* saliency maps is $2l\cdot O\left(M^{2}\log_{2}M\right)$. The computational complexity of the accumulation and decision for all the saliency maps is $\left(l-1\right)\cdot O\left(M^{2}\right)+l\cdot O\left(M^{2}\right)$, and for discriminating operation and ROIs imaging is $O\left(M^{2}\right)+p\cdot O\left(\left(M-K\right)q^{2}\right)$. The total computational complexity of the proposed SAR processing approach is calculated by

(15) $$\begin{array}{cc} T & =O\left(KM^{2}\right)+2l\cdot O\left(M^{2}\log_{2}M\right)+\left(l-1\right)\cdot O\left(M^{2}\right)+l\cdot O\left(M^{2}\right)+O\left(M^{2}\right)+p\cdot O\left(\left(M-K\right)q^{2}\right)\\ & =O\left(KM^{2}\right)+2l\cdot O\left(M^{2}\log_{2}M\right)+2l\cdot O\left(M^{2}\right)+p\cdot O\left(\left(M-K\right)q^{2}\right) \end{array}$$

In most cases, K≫l. Therefore, the total computational complexity of the proposed method is in the order of $O\left(KM^{2}\right)$, which is smaller than the complexity of the most time domain imaging algorithms.

## 3. Experiments and Analysis

**Figure 3:**
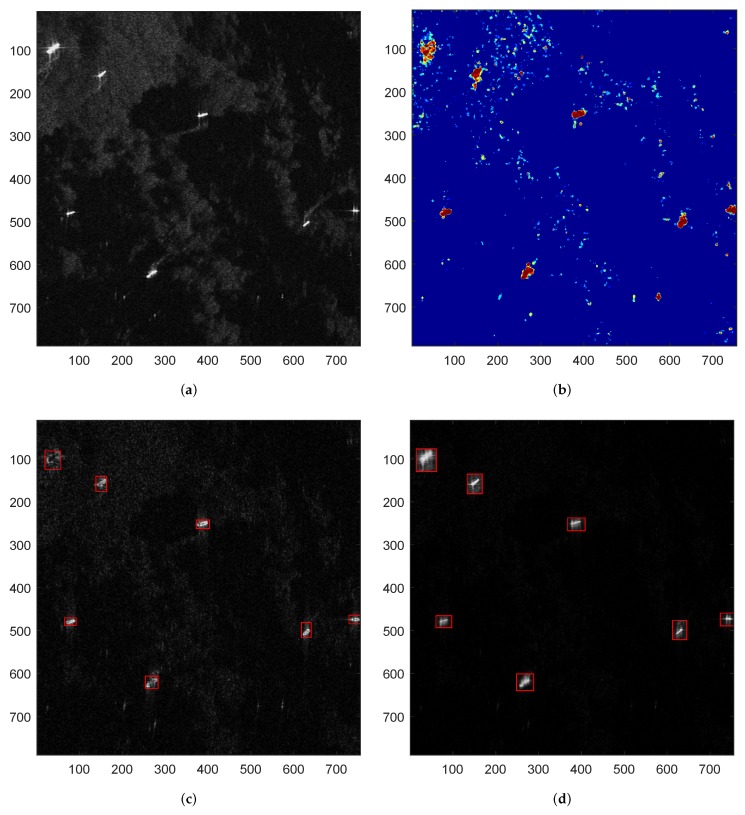
Detection and imaging results of a heterogeneous sea scene by proposed approach. (**a**) original sea scene; (**b**) final accumulated saliency map of proposed approach; (**c**) detection result of proposed approach and (**d**) SAR imaging result of proposed approach.

In this section, the proposed SAR processing approach will be evaluated based on two SAR imaging scenes, namely a heterogeneous sea scene and a complex ground scene, which are shown in Figure 3a and Figure 5a, respectively. The sea scene including seven ships is collected by Sentinel-1 A with 780×755 pixels. The ground scene comes from the Moving and Stationary Target Acquisition and Recognition (MSTAR) [33] clutter dataset with 800×620 pixels. This scene is located near Redstone Arsenal at Huntsville, Alabama, USA. Nine ground targets from MSTAR dataset are embedded on the clutter scene to assess the detection performance of the proposed approach.

In order to simulate the whole process of the proposed approach, the SAR echoes are generated with those two imaging scenes under spotlight pattern. Then the proposed SAR processing approach for target detection and imaging will be conducted based on those echoes. The parameters of SAR imagery formation and visual saliency detection in our method are set as follows. The velocity of the platform is 100 mmss, the flight height is 2000 m, the center frequency is 5 GHz, and the bandwidth is 300 GHz. The range resolution and the full aperture resolution at the cross range both are 0.5 m. The cross range resolution of the first intermediate SAR image $I_{1}\left(x,r\right)$ for visual saliency detection is 2 m, and the resolution difference between two successive intermediate SAR image is 0.2 m. The weight coefficients in the experiments are set as $\omega_{i}=i,$ i=1,2,⋯, *N*, and the threshold parameter δ is 0.707, taking the tradeoff between the missing and false alarm into consideration.

In the experiments, the detection and ROIs imaging results of the sea and ground scenes will be illustrated by the proposed SAR processing approach. Besides, the detection performance of the proposed approach will be compared with two other methods, CFAR method based on the $G^{0}$ distribution [34] and the variance weighted information entropy (VWIE) method [35], which are representative methods in SAR target detection. Finally, the detection performance of these methods are analyzed.

### 3.1. Experimental Results

Figure 3 shows the detection and imaging results of the proposed SAR processing approach under a heterogeneous sea background. In these sub-figures, the red rectangle denotes the correct detection or imaging result, and the green rectangle means the false alarms. Figure 3a is the original heterogeneous sea scene containing seven ships. Figure 3b is the final accumulated saliency map of the proposed approach, and Figure 3c,d present the final detection and imaging results of the proposed SAR processing approach, respectively.

From Figure 3, we can see that the proposed SAR processing approach can not only accurately detect the ship targets, but also generate high resolution image chips of ROIs, which realizes simultaneous target detection and image formation.

**Figure 4:**
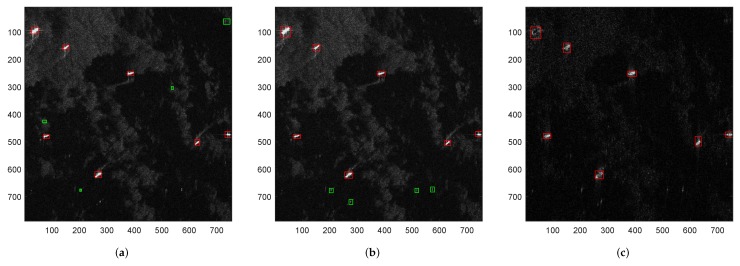
Detection results of a heterogeneous sea scene by various methods. (**a**) detection result of CFAR method; (**b**) detection result of VWIE method and (**c**) detection result of proposed approach.

Now we will test the detection performances of CFAR, VWIE and the proposed approach. Figure 4 illustrates the detection results of the three methods. As we all know, CFAR and VWIE are two representative SAR detection methods, and they must be carried out after the full aperture SAR imagery formation. Hence, the detection results of CFAR and VWIE in Figure 4 are based on the high-resolution SAR images, and the result of the proposed approach comes from the sub-aperture SAR image.

From Figure 4, it can be seen that although the CFAR and VWIE methods can find out the targets from the sea scene, these two target detection methods lead to different degrees of false alarms. In contrast, the proposed SAR processing approach can accurately detect the ship targets from the low resolution imagery without false or missing alarms.

**Figure 5:**
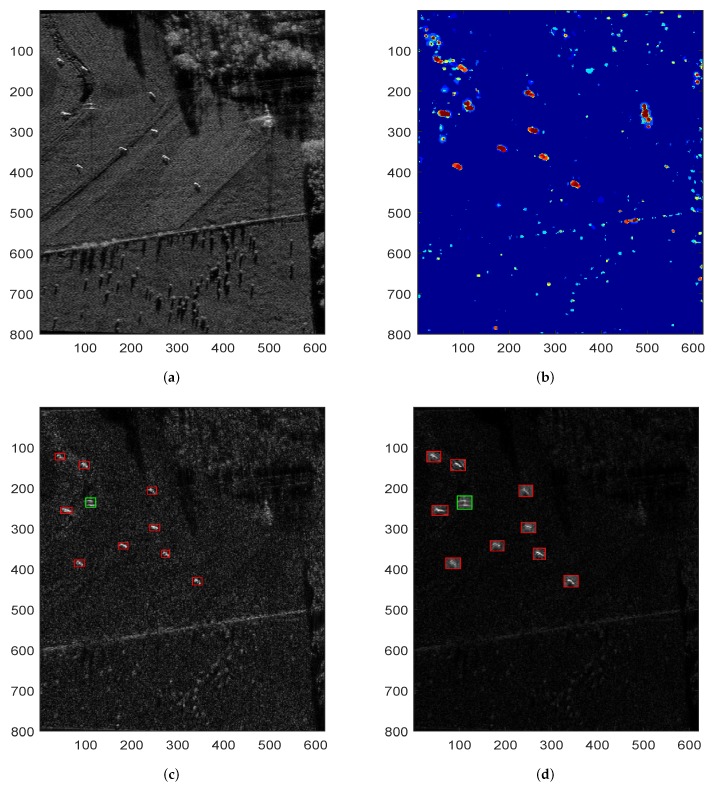
Detection and imaging results of a complex ground scene by proposed approach. (**a**) original ground scene; (**b**) final accumulated saliency map of proposed approach; (**c**) detection result of proposed approach and (**d**) SAR imaging result of proposed approach.

**Figure 6:**
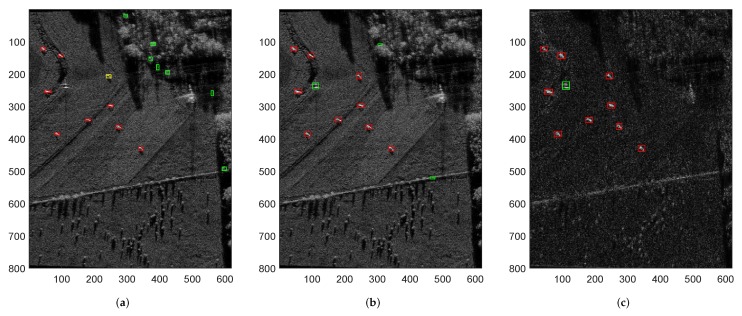
Detection results of a complex ground scene by various methods. (**a**) detection result of CFAR method; (**b**) detection result of VWIE method and (**c**) detection result of proposed approach.

Figure 5 shows the detection and imaging results of the proposed SAR processing approach under a complex ground scene, where the red rectangle denotes the correct detection or imaging result, the green rectangle means the false alarms, and the yellow rectangle represents the neglect of target. Figure 5a is the original complex ground scene containing nine vehicles. Figure 5b is the final accumulated saliency map of the proposed approach, and Figure 5c,d show the final detection and imaging results of the proposed SAR processing approach, respectively. Figure 6 shows the detection results of CFAR, VWIE methods and the proposed SAR processing approach, respectively. Just like Figure 4, the detection results of CFAR and VWIE come from the high-resolution SAR images, and the result of the proposed method is based on the sub-aperture SAR image.

From Figure 5 and Figure 6, we can see that CFAR method has some false alarms on natural clutter regions while with a neglected target. There is no missing target on VWIE detection result, however, it still has three false alarms. In contrast, the proposed SAR processing approach can find out all the ground targets only with one false alarm. It also can get full aperture integration for the ROIs, which is beneficial to the following image interpretation or ATR.

### 3.2. Performance Analysis

In this subsection, we utilize figure-of-merit (FoM) [36] to quantitatively evaluate the detection performances of the proposed approach and other two methods. The FoM of the detection result can be calculated by

(16) $$FoM=\frac{M_{d}}{M_{fa}+M_{t}}$$

where $M_{d}$ is the number of correct detections, $M_{fa}$ denotes the number of false alarms and $M_{t}$ is the number of real targets on the scene. A large value of the FoM means the method is of a good target detection performance.

The number of correct detections, false alarms, real targets on the two scenes, and the corresponding FoMs of the detection results for the three methods are listed in Table 1. From Table 1, it can be seen that while the FoMs of all the detection methods are more than 0.5, the performances of those methods are different. The FoM of the proposed approach is higher than the other two methods, which means the proposed approach performs much better than CFAR and VWIE methods.

All the experiments carried out have shown that the proposed approach has a good capability in simultaneous target detection and image formation.

## 4. Conclusions

In this paper, a novel SAR processing approach is proposed for simultaneous target detection and image formation. Inspired by the human visual system, this approach is conducted based on the time domain SAR imaging and visual saliency detection. The multiresolution SAR images are generated by time domain SAR imaging algorithm, and the intermediate saliency maps are detected by the visual saliency process based on those images. The accumulated maps are iteratively generated until the result with a sufficient confidence level. Thus, the target regions are located after some screening operations, and the SAR imagery with high-resolution detected target regions and low-resolution background are obtained. We have carried out extensive experiments, and the results have shown that the proposed approach can accurately detect the target regions from both the sea and ground scenes, and simultaneously obtain the high resolution imaging results of those detected target regions.

## Figures and Tables

**Table 1:** FoMs of detection results for three methods.

	$M_{d}$	$M_{\mathrm{fa}}$	$M_{t}$	**FoM**
Heterogeneous sea scene (Figure 4)				
CFAR method	7	4	7	0.636
Proposed approach	7	0	7	1
Complex ground scene (Figure 6)				
CFAR method	8	7	9	0.500
VWIE method	9	3	9	0.750
Proposed approach	9	1	9	0.900
